# Estimated respiratory arousal threshold in patients with rapid eye movement obstructive sleep apnea

**DOI:** 10.1007/s11325-021-02399-9

**Published:** 2021-05-17

**Authors:** Tetsuro Hoshino, Ryujiro Sasanabe, Kenta Murotani, Reiko Hori, Mamiko Mano, Atsuhiko Nomura, Noriyuki Konishi, Masayo Baku, Yoshitomo Nishio, Chihiro Kato, Wojciech Kuczynski, Toshiaki Shiomi

**Affiliations:** 1 Department of Sleep Medicine and Sleep Disorder Center, Aichi Medical University Hospital, 1-1 Nagakute, Aichi, 4801195 Japan; 2 Biostatistics Center, Graduate School of Medicine, Kurume University, 67 Asahimachi, Kurume, Fukuoka 8300011 Japan; 3 Department of Oral and Maxillofacial Surgery, Aichi Medical University Hospital, 1-1 Nagakute, Aichi, 4801195 Japan; 4 Department of Sleep Medicine and Metabolic Disorders, Medical University of Lodz, 90-001 Lodz, Poland

**Keywords:** Sleep apnea, REM obstructive sleep apnea, Phenotype, Insomnia, Respiratory arousal threshold

## Abstract

**Purpose:**

Rapid eye movement (REM) obstructive sleep apnea (OSA) is a prevalent clinical phenotype. However, the literature focusing on the pathophysiology of REM OSA is limited. This study compared the proportion of individuals with a low respiratory arousal threshold between patients with REM and non-REM OSA.

**Methods:**

REM OSA was defined as having an apnea–hypopnea index (AHI) ≥ 5 and AHI during REM (AHI-REM)/AHI during NREM (AHI-NREM) ≥ 2. REM OSA was sub-divided into REM-predominant OSA and REM-isolated OSA. REM-predominant OSA was defined as satisfying the definition of REM OSA and having an AHI-NREM ≥ 5. REM-isolated OSA was defined as satisfying the definition of REM OSA and having an AHI-NREM < 5. Patients with an AHI-REM/AHI-NREM < 2 were defined as having non-REM OSA. A low respiratory arousal threshold was defined as having 2 or more of the following conditions: AHI < 30 events/h, proportion of hypopnea > 58.3%, and nadir SpO_2_ > 82.5%.

**Results:**

The proportions of individuals with low respiratory arousal thresholds among individuals with REM-predominant OSA and REM-isolated OSA were significantly higher (77.2% and 93.7%, respectively) than that of patients with non-REM OSA (48.6%). This was also true when the analysis was performed according to sex.

**Conclusion:**

These results indicate that a low respiratory arousal threshold might be an important endotype that contributes to the pathogenesis of REM OSA, especially in REM-isolated OSA.

## Introduction

Obstructive sleep apnea (OSA) is a heterogeneous disorder with both upper airway anatomical vulnerability and non-anatomical traits—including impairment in pharyngeal dilator muscle control and function to airway narrowing, respiratory control instability, and low arousal threshold [1]. Identifying the pathophysiological heterogeneity of OSA is essential for precision medicine.

Recent epidemiological studies have showed that rapid eye movement (REM) OSA is a highly prevalent clinical phenotype that affects 12.2–24.6% of patients with OSA and is characterized by apnea and hypopnea events that predominantly or exclusively occur during REM sleep [2]. Although personalized management strategies for OSA have been developed—including anatomical and non-anatomical approaches—few studies address the pathogenesis of REM OSA. This complicates efforts to describe the best treatment method for this entity [3]. Furthermore, REM OSA appears independently associated with adverse cardiovascular, metabolic, and neurocognitive outcomes [4]. Therefore, it is important to elucidate the pathophysiology underlying REM OSA.

Recently, two studies have indicated that REM OSA was associated with depression and insomnia. The authors mentioned that a low respiratory arousal threshold might be an important endotype that contributes to the pathogenesis of REM OSA [5, 6]. Termination of respiratory events is associated with cortical arousal; therefore, respiratory arousal has been considered to be a potentially lifesaving event that prevents asphyxia during sleep [7]. Alternatively, low respiratory arousal thresholds contribute to prevent deeper stages of sleep associated with stable breathing, ventilatory instability, and increase cortical arousal and sleep fragmentation even in cases of mild upper airway narrowing. Repetitive arousals prevent the accumulation of chemical stimuli required to activate the upper airway dilator muscles [8]. Therefore, a low respiratory arousal threshold is an important endotype that contributes to the pathogenesis of OSA.

Currently, invasive procedures using epiglottic or esophageal pressure catheters must identify the respiratory arousal threshold [9]. However, Edwards et al. recently developed a clinical screening tool that identifies patients with OSA having a low respiratory arousal threshold based on three variables obtained from nocturnal polysomnography (PSG). This method correctly predicted a low respiratory arousal threshold in 84% of patients [9]. We used this clinical screening tool to identify individuals with a low respiratory arousal threshold from two groups of patients: those with REM OSA and those with non-REM OSA.

## Methods

### Study population

**Fig. 1:**
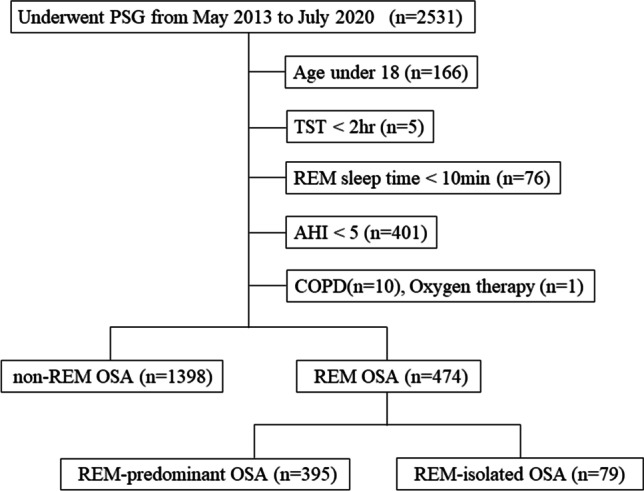
Flow diagram of the study. AHI, apnea–hypopnea index; COPD, chronic obstructive pulmonary disease; OSA, obstructive sleep apnea; PSG, polysomnography; REM, rapid eye movement; TST, total sleep time

This single-center retrospective observational study examined 2531 patients who underwent PSG at the Department of Sleep Medicine of the Aichi Medical University Hospital from May 2013 to July 2020. A total of 659 patients were excluded: 166 patients aged < 18 years, 5 patients who slept for < 2 h during nocturnal PSG, 76 patients who were in REM sleep for < 10 min, 401 patients with an apnea–hypopnea index (AHI) of < 5, 10 patients with chronic obstructive pulmonary disease (COPD), and 1 patient who was receiving oxygen therapy. Ultimately, 1872 adult patients with OSA were enrolled in the study (Fig. 1). At the time of their first visits to our department, the patients were required to complete the Pittsburgh Sleep Quality Index (PSQI) and Epworth Sleepiness Scale (ESS) questionnaires.

### Polysomnography

Nocturnal PSG was performed using the SOMNOscreen plus PSG system (SOMNOmedics, Randersacker, Germany), Alice 5 or 6 System (Respironics Inc., Murrysville, PA, USA), and PSG-1100 (Nihon Kohden Co., Tokyo, Japan). The following physiological parameters during sleep were simultaneously recorded: electro-encephalogram, electro-cardiogram, chin and anterior tibialis electro-myogram, bilateral electro-oculogram, nasal thermistor and prong pressure, respiratory inductance plethysmography bands on chest and abdomen, arterial oxygen saturation, body position, and snoring sound. All polysomnographic parameters were scored manually based on the American Academy of Sleep Medicine Manual for the Scoring of Sleep and Associated Events [10]. OSA was defined as having an AHI ≥ 5 according to the International Classification of Sleep Disorders second edition criteria [11].

A low respiratory arousal threshold was defined as having two or more of the following conditions: AHI < 30 events/h, proportion of hypopnea > 58.3%, and nadir SpO_2_ > 82.5% [9].

### Definition of REM OSA, REM-predominant OSA, and REM-isolated OSA

There was no uniform clinical definition for REM OSA at the time of this study. In reference to previous reports, REM OSA was defined as having an AHI ≥ 5 and AHI-REM/AHI-NREM ≥ 2 [3]. Moreover, we sub-classified REM OSA as follows:

• REM-isolated OSA: AHI ≥ 5, AHI-REM/AHI-NREM ≥ 2, and AHI-NREM < 5.

• REM-predominant OSA: AHI ≥ 5, AHI-REM/AHI-NREM ≥ 2, and AHI-NREM ≥ 5.

Patients with AHI-REM/AHI-NREM < 2 were defined as having non-REM OSA.

### Statistical analysis

Continuous variables were expressed as medians and interquartile ranges. Comparison of polysomnographic and demographic features between patients with REM OSA and non-REM OSA was conducted using the Mann–Whitney *U* test. Comparison between categorical variables was conducted using the Chi-square test. A multivariate logistic regression model was used to investigate the odds ratio with a 95% confidence interval of patients with REM OSA for developing a respiratory arousal threshold score of ≥ 2 after adjusting for medication, including hypnotics, antidepressants, and antipsychotics. Statistical analyses were performed using SAS 9.4 (SAS Institute Inc., Cary, NC, USA).

## Results

Among the 1872 patients, 474 had REM OSA, and 1398 had non-REM OSA. Among patients with REM OSA, 395 patients satisfied the definition of REM-predominant OSA, and 79 patients satisfied the definition of REM-isolated OSA.

Table 1 shows the comparison of patients’ characteristics and polysomnographic variables between REM OSA and non-REM OSA patients. Compared to patients with non-REM OSA, patients with REM OSA had a significantly higher PSQI and percentage of REM sleep/total sleep time (TST). In addition, the percentage of individuals satisfying each component of the respiratory arousal threshold score and those using hypnotic, antidepressant, and antipsychotic medications were higher in the REM OSA compared to the non-REM OSA group.

**Table 1:** Comparison of patients’ characteristics and polysomnographic variables between REM OSA and non-REM OSA patients

	REM OSA (*n* = 474)	non-REM OSA (*n* = 1398)	*p* Value
Age (years)	57 (46, 69)	58 (48, 69)	0.051
Male (%)	275 (58.0)	1131 (80.9)	< .001*
BMI (kg/m^2^)	25.1 (22.5, 28.8)	25.0 (22.6, 28.4)	0.861
ESS (points)	7 (4, 11)	8 (5, 12)	0.027*
PSQI (points)	7 (5, 12)	6 (4, 9)	< .001*
TST (min)	455.0 (433.9, 487.1)	460.5 (439.0, 488.0)	0.030*
Percentage of REM sleep/TST (%)	16.9 (12.1, 21.3)	15.8 (11.7, 20.3)	0.009*
Percentage of N1 sleep/TST (%)	27.6 (18.6, 41.0)	46.4 (32.7, 63.9)	< .001*
Percentage of N2 sleep/TST (%)	52.7 (41.2, 61.9)	36.0 (20.4, 48.1)	< .001*
Percentage of N3 sleep/TST (%)	0 (0, 0.3)	0 (0, 0.1)	0.278
AHI (events/h)	15.8 (9.0, 23.5)	36.5 (19.3, 57.2)	< .001*
AHI-REM (events/h)	38.0 (25.7, 54.0)	35.5 (14.3, 54.5)	< .001*
AHI-NREM (events/h)	10.9 (5.9, 18.0)	36.8 (19.8, 57.8)	< .001*
Nadir SpO_2_ (%)	84.0 (79.0, 88.0)	82.0 (74.0, 87.0)	< .001*
CT90 (%)	0.6 (0.1, 2.1)	1.6 (0.1, 8.1)	< .001*
PLMI (events/h)	0 (0, 10.1)	0 (0, 7.0)	0.052
ArI (events/h)	23.5 (17.3, 31.7)	39.0 (27.5, 55.7)	< .001*
Components of arousal threshold score			
AHI < 30 events/h (%)	419 (88.4)	552(39.5)	< .001*
Proportion of hypopneas > 58.3 (% of all events)	365 (77.0)	746 (53.4)	< .001*
Nadir SpO2 > 82.5% (%)	274 (57.8)	658 (47.1)	< .001*
Proportion with arousal threshold score ≥ 2 (%)	379 (80.0)	639 (48.6)	< .001*
Male (%)	215 (78.2)	496 (43.9)	< .001*
Female (%)	164 (82.4)	143 (53.6)	< .001*
Medication			
Hypnotics (%)	164 (34.6)	267 (19.1)	< .001*
Antidepressants (%)	46 (9.7)	71 (3.7)	0.001*
Antipsychotics (%)	27 (5.7)	35 (2.5)	0.002*

Continuous variables are expressed as medians and interquartile ranges (25th percentiles, 75th percentiles). Categorical variables are expressed as numbers (percentages). *p < 0.05

*AHI* apnea–hypopnea index, *ArI* arousal index, *BMI* body mass index, *ESS* Epworth Sleepiness Scale, *N1* sleep stage 1, *N2* sleep stage 2, *N3* sleep stage 3, *OSA* obstructive sleep apnea, *PSQI* Pittsburgh Sleep Quality Index, *REM* rapid eye movement, *SpO*_*2*_ peripheral capillary oxygen saturation, *TST* total sleep time

Table 2 shows the comparison of patients’ characteristics and polysomnographic variables between REM-predominant OSA and REM-isolated OSA patients. Compared to patients with REM- predominant OSA, patients with REM-isolated OSA showed a significantly higher PSQI and nadir SpO_2_. The percentage of individuals satisfying components of the respiratory arousal threshold score (including AHI < 30 events/h and nadir SpO2 > 82.5%) and those using hypnotics and antidepressants were higher in the REM-isolated OSA compared to the REM-predominant OSA group.

**Table 2:** Comparison of patients’ characteristics and polysomnographic variables between REM-predominant OSA and REM-isolated OSA patients

	REM-predominant OSA (*n* = 395)	REM-isolated OSA (*n* = 79)	*p* Value
Age (years)	57 (46, 70)	58 (44, 69)	0.568
Male (%)	236 (59.7)	39 (49.4)	0.104
BMI (kg/m^2^)	25.6 (22.8, 29.3)	23.4 (21.5, 25.8)	< .001*
ESS (points)	8 (5, 11)	6 (4, 11)	0.115
PSQI (points)	7 (5, 11)	9 (6, 13.5)	0.030*
TST (min)	455.0 (433.0, 487.0)	457.0 (441.0, 493.5)	0.536
Percentage of REM sleep/TST (%)	16.8 (12.1, 20.8)	17.4 (12.0, 23.2)	0.165
Percentage of N1 sleep/TST (%)	29.1 (19.4, 42.0)	21.0 (14.6, 34.2)	< .001*
Percentage of N2 sleep/TST (%)	51.9 (39.4, 61.3)	57.2 (49.1, 65.7)	< .001*
Percentage of N3 sleep/TST (%)	0 (0, 0.3)	0 (0, 0.2)	0.800
AHI (events/h)	17.6 (11.5, 25.5)	6.8 (5.9, 7.9)	< .001*
AHI-REM (events/h)	42.1 (29.1, 55.6)	22.3 (17.7, 33.4)	< .001*
AHI-NREM (events/h)	12.9 (8.1, 19.4)	3.8 (2.9, 4.2)	< .001*
Nadir SpO_2_ (%)	83.0 (78.0, 87.0)	88.0 (84.0, 91.0)	< .001*
CT90 (%)	0.8 (0.1, 2.3)	0.1 (0, 0.5)	< .001*
PLMI (events/h)	0 (0, 10.4)	0 (0, 9.2)	0.973
ArI (events/h)	24.5 (18.1, 32.1)	18.8 (13.7, 25.1)	< .001*
Components of arousal threshold score			
AHI < 30 events/h (%)	340 (86.1)	79 (100)	< .001*
Proportion of hypopneas > 58.3 (% of all events)	306 (77.5)	59 (74.7)	0.660
Nadir SpO2 > 82.5% (%)	208 (52.7)	66 (83.5)	< .001*
Proportion with arousal threshold score ≥ 2 (%)	305 (77.2)	74 (93.7)	< .001*
Male (%)	179 (75.8)	36 (92.3)	0.021*
Female (%)	126 (79.2)	38 (95.0)	0.019*
Medication			
Hypnotics (%)	126 (31.9)	38 (48.1)	0.007*
Antidepressants (%)	31 (7.8)	15 (19.0)	0.006*
Antipsychotics (%)	19 (4.8)	8 (10.1)	0.104

Continuous variables are expressed as medians and interquartile ranges (25th percentiles, 75th percentiles). Categorical variables are expressed as numbers (percentages). *p < 0.05

*AHI* apnea–hypopnea index, *ArI* arousal index, *BMI* body mass index, *ESS* Epworth Sleepiness Scale, *N1* sleep stage 1, *N2* sleep stage 2, *N3* sleep stage 3, *OSA* obstructive sleep apnea, *PSQI* Pittsburgh Sleep Quality Index, *REM* rapid eye movement, *SpO*_*2*_ peripheral capillary oxygen saturation, *TST* total sleep time

**Fig. 2:**
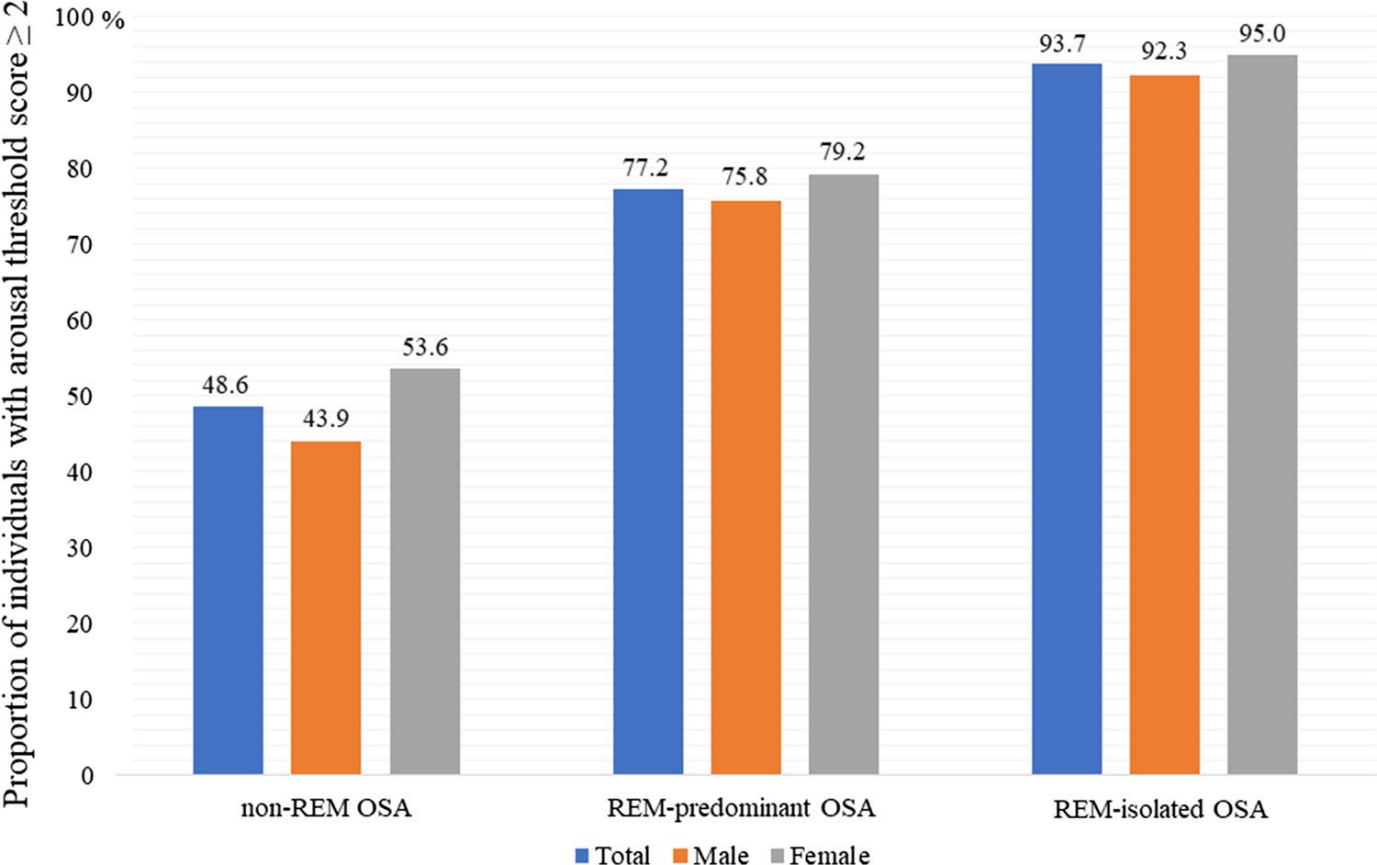
Proportion of individuals with a low arousal threshold score ≥ 2. OSA, obstructive sleep apnea; REM, rapid eye movement

Significantly, more individuals with REM-predominant OSA and REM-isolated OSA demonstrated low respiratory arousal thresholds (77.2% and 93.7%, respectively) compared to patients with non-REM OSA (48.6%). With respect to male patients, the proportions of individuals with low respiratory arousal thresholds among patients with REM-predominant OSA and REM-isolated OSA were significantly higher (75.8% and 92.3%, respectively) than that among patients with non-REM OSA (43.9%) (Fig. 2). The same finding was true for females; the proportions of individuals with low respiratory arousal thresholds among patients with REM-predominant OSA and REM-isolated OSA were significantly higher (79.2% and 95.0%, respectively) than patients with non-REM OSA (53.6%).

Table 3 shows the results of multivariate logistic regression analysis for the association between REM OSA and having a respiratory arousal threshold score of ≥ 2. In the unadjusted model, REM-predominant OSA (crude OR, 4.03; 95% CI, 3.11–5.21; *p* < 0.001) and REM-isolated OSA (crude OR, 17.57; 95% CI, 7.06–43.73) were significantly associated with developing an arousal threshold score of ≥ 2. In the model adjusted for medications including hypnotics, antidepressants, and antipsychotics, REM-predominant OSA (adjusted OR, 3.94; 95% CI, 3.03–5.10; *p* < 0.001) and REM-isolated OSA (adjusted OR, 16.57; 95% CI, 6.62–41.45) were significantly associated with developing an arousal threshold score of ≥ 2.

**Table 3:** Association between REM OSA and respiratory arousal threshold score ≥ 2

	Crude OR (95% CI)	*p* Value	Adjusted OR (95% CI)	*p* Value
Non-REM OSA	1 [Reference]		1 [Reference]	
REM-predominant OSA	4.03 (3.11–5.21)	*p* < .001*	3.94 (3.03–5.10)	*p* < .001*
REM-isolated OSA	17.57 (7.06–43.73)	*p* < .001*	16.57 (6.62–41.45)	*p* < .001*

*OSA* obstructive sleep apnea, *REM* rapid eye movement, *OR* odds ratio, *CI* confidence interval. *p < 0.05

## Discussion

In a previous study conducted at our institute, we found that insomnia, rather than daytime sleepiness, was an important complaint in patients with REM OSA [6]. Although the causal relationships have not been established, the previous study indicated a strong association between insomnia and low arousal thresholds [12]. Therefore, we conducted the current study focusing on the respiratory arousal threshold in patients with REM OSA.

All patients with OSA have some degree of upper airway vulnerability. A previous study found that 36% of patients with OSA had impairment in pharyngeal dilator muscle control and function to airway narrowing, 36% had respiratory control instability, and 37% had a low arousal threshold [1]. Our study showed that more than 90% of patients with REM-isolated OSA had a low respiratory arousal threshold. Zinchuk et al. found that non-obese patients with a low respiratory arousal threshold had worse adherence to CPAP therapy [13]. If, as we hypothesized, a low respiratory arousal threshold is a significant endotype of REM OSA, then this finding would be consistent with previous studies that reported low adherence to CPAP therapy among patients with REM OSA [14, 15]. Randomized controlled trials have successfully demonstrated that eszopiclone, zopiclone, and zolpidem increase respiratory arousal thresholds [3, 8]. Therefore, the use of these drugs may improve CPAP therapy adherence in patients with REM OSA.

Patients with REM OSA were significantly more likely to take hypnotics and antidepressants, which are well known to increase the arousal threshold [3]. Patients with REM OSA have more symptoms of insomnia and depression, rather than daytime sleepiness [5, 6]. Therefore, we assume that many are prescribed hypnotics or antidepressants before a sleep examination. Furthermore, after adjusting for the medication including hypnotics, antidepressants, and antipsychotics, the multivariate logistic regression model showed that REM-predominant OSA (adjusted OR, 3.94; 95% CI, 3.03–5.10; *p* < 0.001) and REM-isolated OSA (adjusted OR, 16.57; 95% CI, 6.62–41.45) were significantly associated with developing an arousal threshold score of ≥ 2. These results indicate that pharmacotherapy targets that increase the respiratory arousal threshold might be effective for patients with REM OSA, especially in REM-isolated OSA.

Our results should be interpreted with caution. The method for scoring the respiratory arousal threshold was an indirect estimation. Therefore, our results need to be verified using direct measurements of the respiratory arousal threshold.

In conclusion, compared with patients with non-REM OSA, significantly more patients with REM OSA demonstrated low respiratory arousal thresholds. A low respiratory arousal threshold may be an important endotype that contributes to the pathogenesis of REM OSA, especially in patients with REM-isolated OSA. Further studies are necessary to continue the development of personalized management strategies for patients with REM OSA.
